# Hardware Architecture and Cutting-Edge Assembly Process of a Tiny Curved Compound Eye

**DOI:** 10.3390/s141121702

**Published:** 2014-11-17

**Authors:** Stéphane Viollet, Stéphanie Godiot, Robert Leitel, Wolfgang Buss, Patrick Breugnon, Mohsine Menouni, Raphaël Juston, Fabien Expert, Fabien Colonnier, Géraud L'Eplattenier, Andreas Brückner, Felix Kraze, Hanspeter Mallot, Nicolas Franceschini, Ramon Pericet-Camara, Franck Ruffier, Dario Floreano

**Affiliations:** 1 Aix-Marseille Université, CNRS, UMR 7287 ISM, 13288 Marseille, France; E-Mails: raphael.juston@univ-amu.fr (R.J.); fabien.expert@gmail.com (F.E.); fabien.colonnier@univ-amu.fr (F.C.); nicolas.franceschini@univ-amu.fr (N.F.); franck.ruffier@univ-amu.fr (F.R.); 2 Aix-Marseille Université, CNRS, UMR 7346 CPPM, 13288 Marseille, France; E-Mails: godiot@cppm.in2p3.fr (S.G.); breugnon@cppm.in2p3.fr (P.B.); menouni@cppm.in2p3.fr (M.M.); 3 Fraunhofer Institute for Applied Optics and Precision Engineering, 07745 Jena, Germany; E-Mails: robert.leitel@iof.fraunhofer.de (R.L.); wolfgang.buss@iof.fraunhofer.de (W.B.); andreas.brueckner@iof.fraunhofer.de (A.B.); felix.kraze@iof.fraunhofer.de (F.K.); 4 Laboratory of Intelligent Systems, École Polytechnique Fédérale de Lausanne, CH-1015 Lausanne, Switzerland; E-Mails: geraud.leplattenier@epfl.ch (G.L.E.); ramon.pericet@epfl.ch (R.P.-C.); dario.floreano@epfl.ch (D.F.); 5 Laboratory of Cognitive Neuroscience, Department of Biology, University of Tübingen, 72076 Tübingen, Germany; E-Mail: hanspeter.mallot@uni-tuebingen.de

**Keywords:** compound optics, optical sensors, active pixel sensors, flexible electronics, fast bus, robotics

## Abstract

The demand for bendable sensors increases constantly in the challenging field of soft and micro-scale robotics. We present here, in more detail, the flexible, functional, insect-inspired curved artificial compound eye (CurvACE) that was previously introduced in the Proceedings of the National Academy of Sciences (PNAS, 2013). This cylindrically-bent sensor with a large panoramic field-of-view of 180° × 60° composed of 630 artificial ommatidia weighs only 1.75 g, is extremely compact and power-lean (0.9 W), while it achieves unique visual motion sensing performance (1950 frames per second) in a five-decade range of illuminance. In particular, this paper details the innovative Very Large Scale Integration (VLSI) sensing layout, the accurate assembly fabrication process, the innovative, new fast read-out interface, as well as the auto-adaptive dynamic response of the CurvACE sensor. Starting from photodetectors and microoptics on wafer substrates and flexible printed circuit board, the complete assembly of CurvACE was performed in a planar configuration, ensuring high alignment accuracy and compatibility with state-of-the art assembling processes. The characteristics of the photodetector of one artificial ommatidium have been assessed in terms of their dynamic response to light steps. We also characterized the local auto-adaptability of CurvACE photodetectors in response to large illuminance changes: this feature will certainly be of great interest for future applications in real indoor and outdoor environments.

## 1. Introduction

The compound eyes of insects and crustaceans, which show an extraordinarily wide range of designs (see [1] for a review), a remarkable optical layout, high sensitivity in dim light, even at night, and polarized light sensitivity, provide an endless source of inspiration for designing the curved, flexible visual sensors of the future. The paper extends the description of a flexible functional insect-inspired curved artificial compound eye (CurvACE) that was previously introduced by Floreano *et al.* [2]. In particular, we thoroughly describe here the innovative VLSI sensing layout, the accurate assembly fabrication process, the innovative, fast read-out interface of the sensor, as well as the auto-adaptive dynamic response of the CurvACE photodetectors. Several attempts have been recently made to realize miniature compound eyes. Artificial compound planar eyes based on micro-lens arrays with adjustable optical axes have been developed and interfaced with conventional flat CMOS imagers ([3,4]). However, the optical field of view (FOV) was restricted to a range of ± 30°. To overcome this limitation, micro-optics on curved substrates have been introduced using several methods ([5–7]), including the use of spherical bulk lenses [6], planar wide angle and telescopic lenses ([7,8]), micro-prism arrays [9], a tiny flexible camera array [10] and an origami assembly of 2D optic flow sensors [11]. Small omnidirectional cameras ([12–17]) have been designed for robotic applications. However, correcting the distortion of the optics requires large computational resources and the use of classical imagers restricts the frame rate (e.g., 80 frames per second [15]). The challenge of designing an extremely compact eye with a panoramic visual field can only be met by considering not only the optics, but the whole imaging system. Flexible image sensors inspired by the shape of the retina in the vertebrate single lens eye ([18,19]) and curved optoelectronic cameras with a limited field of view were recently presented ([20–22]). In one case, Song *et al.* [23] succeeded in bending a stretchable planar compound eye to obtain a spherical compound eye composed of 180 ommatidia with an overall field-of-view of 160° × 160°. This remarkable design is based on cutting-edge technologies, such as flexible micro-lens arrays and micro-electronics (photodiode), encapsulated within a flexible polyimide support. However, this new principle was associated with non-uniform visual sampling, and because of the very coarse resolution of the eye (the interommatidial angle ranged from 8° to 11°), large computational resources were required to put (“stitch”) together the various subimages acquired by the ommatidia. The state-of-the-art technologies based on planar assembly and the use of rigid materials, such as silicon (VLSI chips) and glass (optics), have been applied to obtain a miniature curved compound eye. Moreover, the presented concept allows for the construction of manifold and even extensive bendable multi-channel sensor shapes. In order to enhance the sensing abilities of future robotic platforms, it was proposed to develop a small, lightweight, power-efficient artificial compound eye endowed with an adaptation mechanism right at the photodetector level, which is able to compensate for considerable changes in the ambient light. Visual sensors must be able to deal with the large dynamic range of natural irradiance levels, which can cover approximately up to nine decades during the course of the day. Animal retinae have partly solved this crucial problem, since their photoreceptors implement a light-adaptation mechanism ([24–26]). As described in Section 3, we equipped each CurvACE photodetector with a neuromorphic adaptation circuit [27], which acts within each ommatidium independently of its 629 neighbors. In addition to fast auto-adaptive photodetectors, a fast communication interface was also included, which was able to read the 42 columns of 15 ommatidia at a very high speed (up to 1950 frames per second). Section 2 summarizes the CurvACE features. Section 3 describes in detail the hardware architecture and the patented method [28] used to construct the CurvACE sensor. Section 4 gives results of tests performed on the compound eye in terms of its photodetectors' responses and its auto-adaptive abilities to various lighting conditions.

## 2. Overview of a Miniature Cylindrical Compound Eye

**Figure 1.**
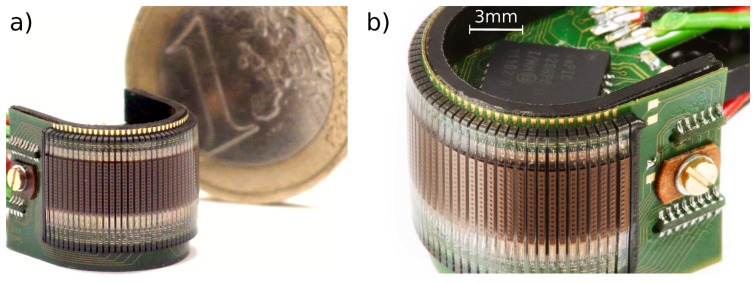
(**a**) Image of the curved artificial compound eye (CurvACE) sensor, which has a radius of curvature of only 6.35 mm, a mass of only 1.75 g and a power consumption of 0.9 W. The cylindrical shape of the eye was obtained by bending a rectangular array consisting of 42 columns of 15 artificial ommatidia (microlens diameter = 172 *μm*). (**b**) The resulting concavity was used to house two printed-circuit-boards carrying two microcontrollers, a rate-gyro, a three-axis accelerometer and other electronic components.

The CurvACE compound eye presented in Figure 1 consists of an ommatidial patch, which has been cylindrically bent and attached to the outside of a curved polymeric scaffold. The ommatidial patch has 630 photodetectors grouped in 42 identical columns of 15 photodetectors (each equipped with its own microoptical lens) which are placed on a thin flexible printed circuit board (PCB) wrapped around the cylindrical scaffold. Interestingly, the whole patch of 630 ommatidia (total thickness 0.85 mm) weighs no more than 0.36 g. Two rigid PCBs bearing signal processing units, which are responsible for the readout of the visual data, have been placed in the concavity space and connected to an external processing unit. Two additional inertial sensors (a three-axis rate gyro and a three-axis accelerometer) were implemented to provide complementary information to visual data. Thanks to this compact mechanical design, the entire device has a volume of only 2.2 cm^3^ and weighs only 1.75 g. The maximum power consumption of the device is 0.9 W. The readout is performed using a direct serial communication bus that acquires data in parallel from the ommatidial columns. The visual data provided by the columns are serialized at a clock frequency of 1 MHz. The visual data are delivered at a maximum frame rate of about 1950 frame per second (fps), which is suitable for fast optic flow (OF) extraction.

## 3. Description of the Flexible Ommatidial Patch

### 3.1. Layout of the Photodetector Layer

**Figure 2.**
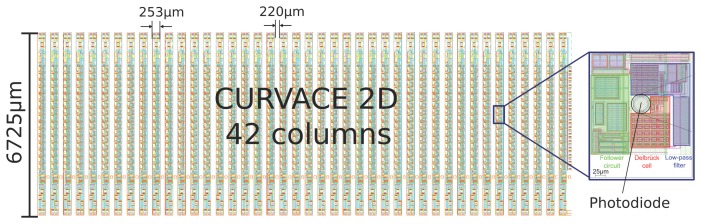
Layout of the sensor die fabricated by means of a CMOS 0.35 *μm* process (XFAB opto option).

**Figure 3.**
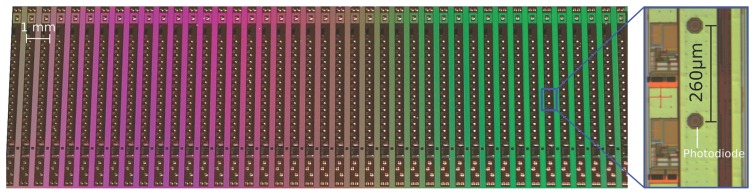
Image of the sensor die (see also the layout in Figure 2) having 42 independently-working columns comprising 15 photodetectors (pixels) each. As shown in the magnified view of one part of the column, the pitch between two octagonal photodetectors is 260 *μm*.

The photodetector array was generated by 0.35 *μm* CMOS technology with an optoelectronic option (XFAB opto option) giving openings with an anti-reflective coating placed above the photodiode areas. Figures 2 and 3 show the layout of the die with 42 columns having a total lot size of 19.845 by 6.765 mm^2^. As part of a multi-project wafer, 132 tested dies were received from a complete 200-mm wafer.

In general, the layout is the same for all columns, except the most right one, which possesses additional readout test pads for each photodetector. As shown in Figure 2 and in the Figure S2 of the Supplementary Data published in [2] , a column consists of 15 photodetectors followed by Delbrück circuits [27], a 16-channel multiplexer (MUX), a 10-bit analog-to-digital converter (ADC), a state-machine and eight electro-static discharge (ESD) shielded pads for wire bonding. An additional bias circuit serves to generate the polarization current required by the self-adaptive and low-pass filter circuits integrated at the photodetector level.

**Figure 4.**
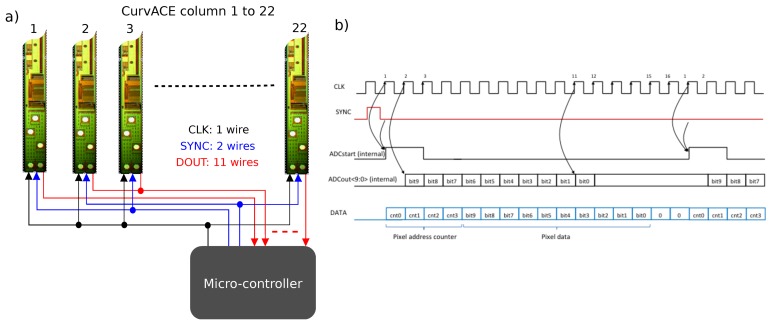
(**a**) Functional scheme of the direct connection protocol (DCP) specially designed to read out each column. With this protocol, a fast parallel readout of the columns is feasible without adding too many wires on the flexible printed circuit board (*i.e.*, keeping the routing simple) (see Figures 10 and 12). To read out 22 columns of CurvACE, this protocol requires only one clock signal (CLK), two synchronization signals (SYNC) and 11 data signals (DOUT) provided by an external micro-controller. An additional micro-controller is therefore required to read out the 20 others (see Figure 5). (**b**) Chronogram of the main signals (CLK, SYNC and DATA) used for the serial readout of each CurvACE column. The microcontroller provides the clock (CLK) and synchronization (SYNC) signals, whereas the data (DATA) lines are read by the micro-controller on each rising edge of the clock.

The main supply voltage was decoupled and divided into one for the analogue circuitry and another for the digital circuitry (four pads). The further processing of the photodiode output signals via a multiplexer (MUX) and the ADC is shown in Figure 4 (three pads for sync, clock and data lines). The remaining pad is an analogue readout of the central photodetector for testing purposes. Moreover, the left (16th) channel of the MUX is reserved for a reference voltage. The width of the self-contained column's circuitry is 253 *μm*, whereas the space to its neighbors is 220 *μm*. These gaps are mandatory for later separation issues and consider clearance areas due to possible damaging of the die. This gives a column pitch of 473 *μm*. Each column contains 15 octagonal Nwell-Psubstrate photodetectors with a diameter of 30 *μm*. The photodiodes are aligned in the column with a vertical spacing of 260 *μm*.

### 3.2. CurvACE Readout Interface

The specifications of the readout interface were very drastic in terms of the number of pads (minimum number of pads per column), bit rate (as fast as possible) and resources available (no flash or Electrically-erasable programmable read-only memory (EEPROM) available for storing an address). Several communication standards were considered for use in CurvACE: the main characteristics of the bus interfaces are summarized in Table 1.

The selected “direct connection protocol” (DCP) is based on:

- a single clock signal sent to each column,
- a sync signal used to start the conversion of the associated columns,
- a digital-out signal (*D_out_*) per column used for the serial transfer of each pixel output signal.

The main advantage of this protocol is that it does not require any addresses (stored in an EEPROM memory, for example) to be able to communicate with a column. Besides, the refresh rate does not depend on the number of columns, and the readout protocol adds only three pads per column, which leads to eight pads in total:

- a digital-out signal (*D_out_*) per column used for the serial transfer of each pixel output signal,
- four pads for the digital (*D_vdd_*) and analog (*A_vdd_* ) power sources and for the ground (*D_Gnd_* and *A_Gnd_*),
- three pads (see Figure 4a) for the serial readout (Clock, Sync, *D_out_*),
- 1 test pad.

At the beginning of each conversion cycle (see Figure 4b), the counter value corresponding to the pixel address is added to the output data. The data is transferred serially through the output shift register during the ADC conversion. Individual bits resulting from the ADC conversion are validated before the end of the ADC conversion. This strategy minimizes the dead time possibly elapsing during the data transfer. At each conversion cycle, 16 bits are sent (four from the counter, 10 from the ADC and two unused bits set at zero) to the external microcontroller. When operating at the maximum sampling frequency of 1 MHz, the ADC therefore provides a maximum sampling rate of about four ksamples/s per photodetector. However, to reduce the number of tracks in the flexible PCB, we decided to use two synchronization signals: one for each group of 21 columns. The maximum sampling rate for reading the 630 ommatidia of the CurvACE sensor was therefore 1950 fps.

**Figure 5.**
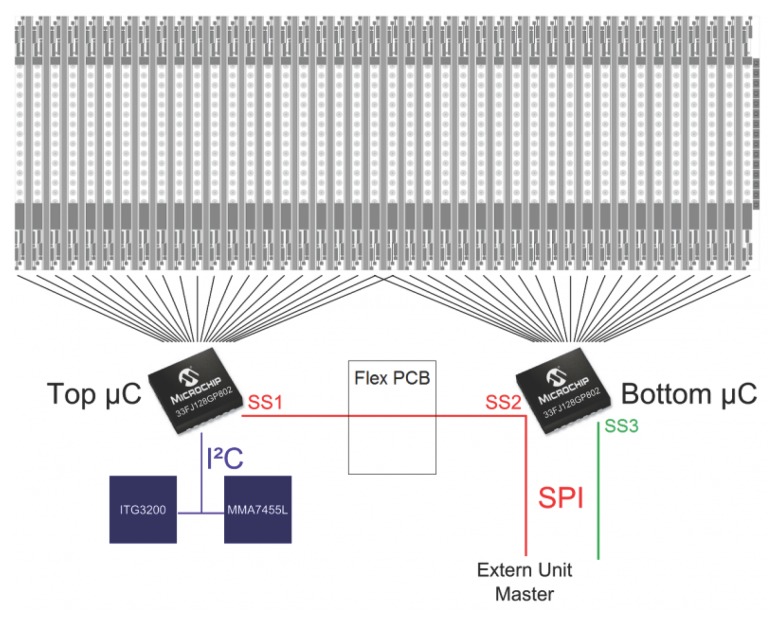
Communication interface between the two microcontrollers (the slaves) and an external processing unit (the master). Two slave-selected signals (SS1–SS2) are used to read the pixel values of the pre-defined regions of interest. The two inertial sensors are addressable by the external processing unit via the top micro-controller.

As shown in Figure 5, two dsPIC33FJ128GP802 microcontrollers from Microchip have been applied for the readout and on-site processing of the data. This microcontroller model has 16 kbyte of RAM, although it measures only 6 by 6 mm^2^. Each micro-controller is responsible for reading out 22 columns (the two central columns were read by both micro-controllers) sequentially by two batches of 11 columns, as we used two synchronization signals. For the readout, a serial direct communication protocol was used. This protocol scans the pixels in every column serially, whereas the columns are read in parallel via 11 data lines connected to each microcontroller (*cf.* Figures 4 and 5). One of the microcontrollers masters the direct connection protocol and generates the clock and the synchronization signals, which are shared by the other for the sake of synchronization. The microcontrollers themselves communicate *via* Serial Peripheral Interface (SPI) bus with an external unit, which can be placed on a robot, as well.

The communication protocol of the SPI interface was designed such that data can be extracted from different ROIs (regions of interest) of the CurvACE sensor specified by the user. Only the pixels corresponding to the chosen regions of interest are transmitted to the external unit. After choosing the number of regions of interest and their size, the protocol allows for the following options:

- frame rate up to the maximal theoretical frame rate of the sensor, namely 1950 fps,
- sampling frequency of the inertial sensor data, which is up to one data per frame,
- number of frames that are sent in two separate 16-bit words: 232 frames can be sent with the same configuration.

### 3.3. Micro-Optics

**Figure 6.**
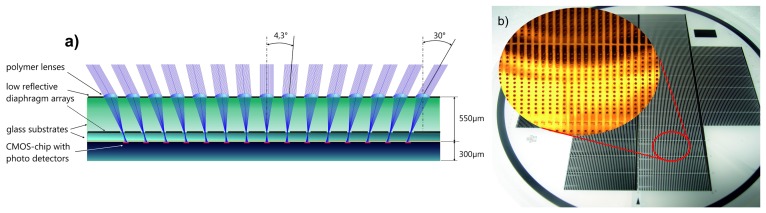
(**a**) Ray tracing (Zemax®) of the optics of a CurvACE column. The central channel is designed for normal incidence, whereas the outer channels register off-axis light incidence of increasing inclination. The outmost channels are designed for a 30° angle of incidence. (**b**) Picture of the micro-optics wafer. Modified from [2].

**Figure 7.**
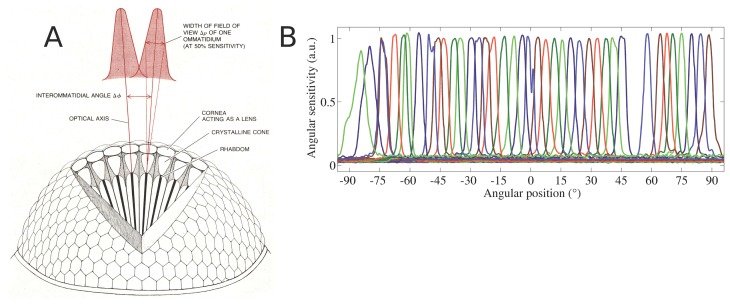
(**A**) Schematic view of an insect's compound eye depicting its two main optical parameters: the interommatidial angle *∆φ*, defined as the angle between optical axes of two adjacent ommatidia, and the acceptance angle ∆*ρ*, defined as the angle at the half width of one ommatidium's angular sensitivity function (ASF). The Gaussian shape of the ASF results from the convolution of the rhabdom with the diffraction figure of the lens. The diameter of the facet lenses in the male blowfly *Calliphora* ranges from 20 *μm* to 40 *μm*, whereas the diameter of the peripheral rhabdomeres is 1.5-2.0 *μm* ([33]). Adapted from ([34]). (**B**) Example of horizontal angular sensitivity functions (ASFs) of each ommatidium measured across the equatorial row (red line) of CurvACE within a range of ±45°. The Gaussian-shaped functions of the CurvACE ASFs were obtained by the defocussing of the microlens and the introduction of a diaphragm (see Figure 8a). The interommatidial angle resulted in an average of *∆φ* of 4.2° ± 0.8° (SD) and an acceptance of ∆*ρ* of 4.2° ± 0.3° (SD). Adapted from [2].

A well-adapted micro-optical system has been mounted onto the photodetector array in order to sample the specified total field of view (FOV) of 180° by 60° by the individual detectors, having a constant acceptance angle of 4.3°. This has been achieved by a multi-aperture approach implying the use of a microlens array [29,30]. To have a gapless registration of FOV, the difference in the chief ray angle between adjacent detectors is set equal to the full-width at half-height (FWHM) of the angular sensitivity function (ASF) of the optical channels. Thus, the ASFs overlap slightly by an intended amount (see Figure 7b). A horizontal FOV of 180° was achieved by the subsequent bending of the assembled sensor device, whereas a vertical FOV of 60° along a single column was obtained by introducing a pitch difference (see Figure 6) between the microlens (pitch of 290 *μm*) and the photodetector arrays (pitch of 260 *μm*). Furthermore, the use of toroidal-shaped microlenses reduces aberrations from off-axis imaging [31]. The micro-optics intends to form a Gaussian intensity distribution at the photodetector plane due to the slight defocussing (see Figure 7b). Such deliberate blurred spots have been observed in natural compound eyes, as well (see Figure 7a) and are beneficial for the detector characteristics and the optical flow processing. As pointed out by Lucas and Kanade [32], smoothing the image suppresses small details (*i.e.*, high spatial frequencies) and, thus, improves the convergence of the algorithm once initialized.

The micro-optics have been fabricated in wafer scale utilizing 100-mm substrates of display glass (Schott, D263T). Microlens masters have been generated by reflow of patterned photoresist and replicated by using highly transparent UV-curable organic-inorganic hybrid material (MRT GmbH, Ormocomp). Besides an aperture defining diaphragm array close to the microlens layer, a second diaphragm array is introduced close to the focal plane in order to prevent crosstalk between neighboring channels and to reduce ghost images. The layers are composed of lithographically patterned low-reflective metal (black chromium). The optical stack has a total thickness of about 570 *μm*, including the sag heights of the microlenses.

### 3.4. Sensor Assembly

**Figure 8.**
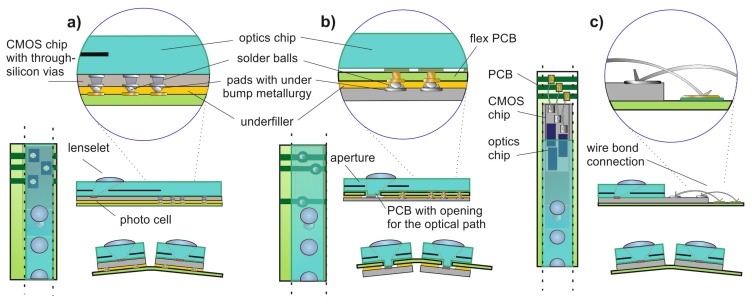
Evaluated assembly concepts: (**a**) Flip chip bonding with backside PCB. This solution requires through silicon vias (TSVs) on the CMOS chips, but would allow for wafer-scale processing. (**b**) Flip chip bonding with the PCB sandwiched between the optics and CMOS chips. This solution implies more complex assembly and PCB layout. (**c**) Wire bonding of the column pads to the flexible PCB. This solution is particularly suiz for small series production.

Since the custom designed CMOS Opto-ASICs have been produced on multi-project wafers, a wafer-level bonding of multi-aperture microoptics and sensors was not an option. Consequently, the optics have been bonded chip-wise by using active alignment techniques. Three different assembling concepts were evaluated for prototype fabrication with the potential for small series production (see Figure 8):

- Flip chip bonding based on through silicon vias (TSVs). This solution, particularly suitable for mass production, has been dropped as a matter of costs and available space (see Figure 8a).
- Flip chip bonding with intermediate flexible PCB. This solution uses conventional top-side electrical contacts, but introduces openings of the PCB located in the middle position between the sensor and optics. Noteworthy, this implied a further adaptation of optics and increased complexity (see Figure 8b).
- Wire bonding of the VLSI chip to the flexible PCB. This solution has been chosen due to the comparatively simple stack assembly of the optics, VLSI chip and flexible PCB. Moreover, wire bonding of dies in multi-chip modules is state-of-the-art, even on flexible PCBs (see Figure 8c).

In the first step, the required components have been prepared for stack mounting. The VLSI and the microoptics chips have been diced from eight-inch and four-inch wafers, respectively. Moreover, the sensor chips have trenching grooves at their backside at the positions of the dicing lanes. These trenches of 100 *μm* in depth and 150 *μm* in width allowed for safe cutting at a later step.

**Figure 9.**
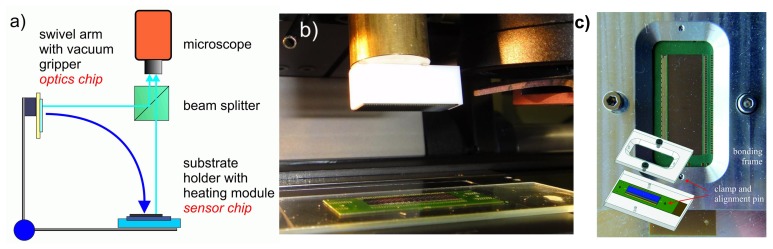
(**a**) Active alignment of the optics and the sensor chips using a Fineplacer^®^ device. (**b**) Adapted vacuum gripper holding microoptics. (**c**) The passive alignment stage for adhesive bonding to the PCB was also suited to wire bonding by applying an additional bonding frame.

The flexible PCBs were fabricated by fine line technology with gold-plated bond pads. As an alignment aid for the positioning of the sensor-optics stacks, a window of the size of the chip has been left free in the solder mask layer. This enables a placement of the stack within a tolerance of ±20 *μm*, which holds for bond pad openings and is required for wire bonding. As shown in Figure 9a,b, the adhesive bonding of the multi-aperture optics and CMOS chip was done by a FINEPLACER^®^ lambda device using active alignment and an adapted vacuum gripper for the former of both [35]. The applied transparent UV-curable adhesive (EPO-TEK^®^OG 146) features high mechanical strength and heat resistance beyond 150 °*C*, which are appropriate for loads during column separation (shear forces) and encapsulation (heat), respectively. The accuracy of positioning (±1.5 *μm*) was better than the one requested by the optic design (±3 *μm*).

In contrast, the mounting of this stack onto the flexible PCB was done passively with a jig aid by means of two-component epoxy glue (EPO-TEK 353ND). Therefore, an adapted assembly stage was used, which allowed for a fast bonding process at an adequate lower level of precision due to a mechanical guide pin (see Figure 9c).

**Figure 10.**
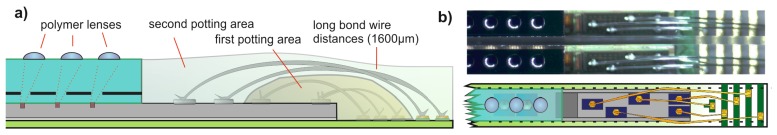
(**a**) Wire bonding scheme for five closely-spaced pads in side view. As five pads cannot be placed side by side, due to the column width, the wire-bonding has been realized in two steps. (**b**) Top view of the realized wire bonding and its scheme.

**Figure 11.**
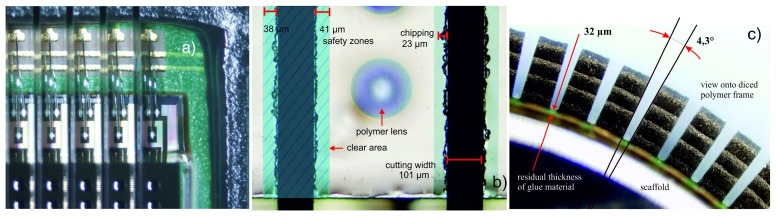
(**a**) Black polymer frame used as a wall for the wire potting. (**b**) Top view of one column after dicing with its safety zones preventing deterioration due to chipping. (**c**) Side view onto the glob-top frame after bending of the flexible sensor array with respect to a pre-defined curvature of a scaffold.

The analog-digital circuit and the readout interface lead to eight interconnection pads per column that are grouped in three and five pads (see Figure 10) at the top and bottom sides, respectively, leading to a total number of 336 interconnections. Due to the column width of 240 *μm*, five pads cannot be placed side by side, and a two-step bonding was performed, which implies an intermediate potting step of the first set of short wires. A final encapsulation covers all bonding wires, but does not spill over the optics. A previously placed polymer frame on the PCB enclosed the area for casting. During wire bonding and encapsulation, a special bonding frame (see Figure 11a) fixed the PCB to the stage and avoided detachments from pulling forces when the bond head lifts.

Finally, precision cut-off by grinding has been applied to separate the sensor array into 42 equal columns of 15 photodetectors (see Figure 11b). Currently, the sawing technology is the most promising way for efficiently cutting the stack of glass, silicon and polymer (glue and glob-top material) of about 870 *μm*. Synthetic-resin-compound diamond saw blades of 100 *μm* in thickness were employed. The backside trenches ensured that the PCB was not touched by the sawing blade and remained intact. Nevertheless, residual glue in the gap between sensor and PCB causes stiffening and impedes its bending. Consequently, an optimized glue thickness is inevitable to prevent uncontrolled breaks and irregular bending. Moreover, it is important to bend it exactly along its designated direction; see Figure 11c. Any torsion may cause a breaking of the columns. Two screws were used to fix the bent PCB to the scaffold of a pre-defined curvature.

## 4. VLSI Implementation and Dynamic Responses of the CurvACE Auto-Adaptive Photodetectors

### 4.1. Description of the VLSI Circuit

The CurvACE photodetector is based on an octagonally-shaped Nwell-Psubstrate photodiode with a diameter of 30 *μm* and on the Delbrück adaptive photodetector cell. It has been shown that a photodiode with an octagonal shape has better sensitivity than a square-shaped one [36]. A low-pass filter next in line limits the cut-off frequency of the photodetector to a value (300 Hz) that is compatible with the sampling rate of the 10-bit ADC used to digitize each photodetector's output signal. The square footage of the circuitry of a single photodetector measures 253 *μm* by 250 *μm*.

**Figure 12.**
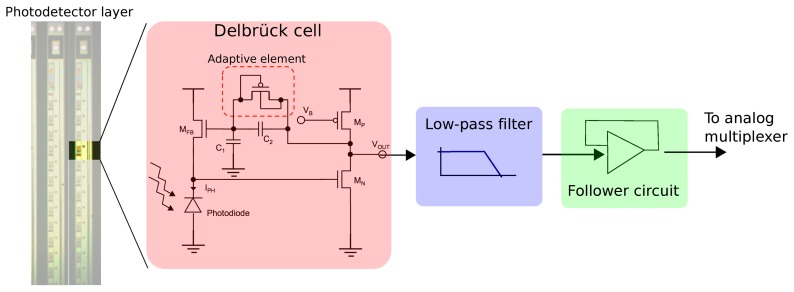
One of the 15 photodetector cell of one CurvACE column. The original circuit developed by Delbrück and Mead [27] was enhanced here by cascading a first-order low-pass filter to prevent temporal aliasing. Adapted from [2].

The Delbrück adaptive photodetector consists of a logarithmic circuit associated with a high gain negative feedback loop, as shown in Figure 12. It is based on a MOSFET feedback (MFB) transistor operating in the sub-threshold region where the current-to-voltage characteristic shows logarithmic variations in a large dynamic range of up to several decades. The adaptive element responsible for the DC output levels acts like a very high resistance and makes the output signal *V_out_* follow the gate voltage of the MFB transistor. The non-linear resistance of the adaptive element decreases in the case of fast transient signals. The adaptation to variations in the ambient light levels is therefore relatively slow, whereas the compensations for changes in contrast are much faster (see Section 4.2).

**Figure 13.**
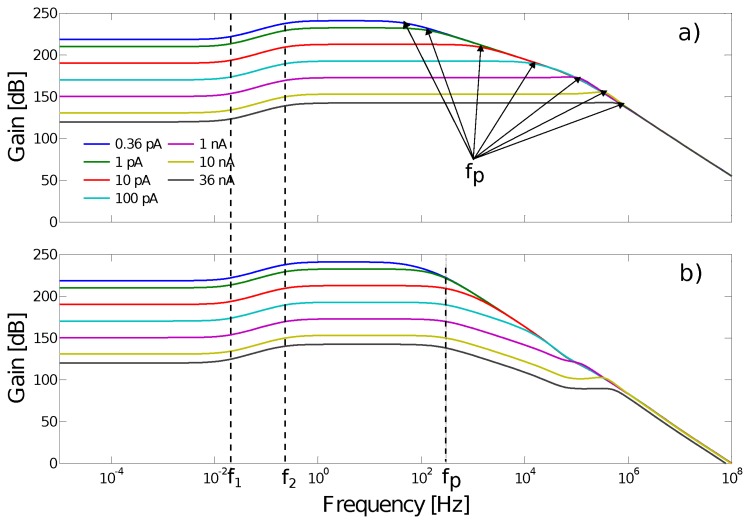
(**a**) Simulated photodetector Bode diagram: the values of the low cut-off frequencies *f*_1_ and *f*_2_ are a function of the resistivity of the adaptive element, whereas the cut-off frequency *f_p_* depends on the value of the photodiode's background current (*I_bg_*). The higher the illuminance is, the higher the frequency *f_p_* will therefore be. (**b**) Bode diagram after the integration of the low-pass filter: the cutoff frequency *f_p_* is kept constant (300 Hz) regardless of the ambient luminosity.

The simulated AC gains obtained with various photodiode background currents are illustrated in Figure 13. The frequencies *f*_1_ and *f*_2_ are those of the adaptive elements with the two time constants τ defined as follows:

(1) $$\tau_{1}=R(C_{1}+C_{2})\;\text{and}\;\tau_{2}=RC_{2}$$

where R is the equivalent resistance of the adaptive element, corresponding to a value of 3.10^12^ Ω in the case of a low input level, *C*_2_ is set at 105 fF, the capacitance ratio is equal to 15 and the frequencies *f*_1_ and *f*_2_ are equal to approximately 0.03 Hz and 0.5 Hz.

The low-pass cut-off frequency (*f_p_*) of the photodetector depends on the background current (*i.e.*, the ambient luminosity). It can be estimated using the following equation:

(2) $$f_{p}=\frac{I_{bg}}{2\pi C_{p}U_{t}}$$

Therefore, *f_p_* varies in a large range from 24 Hz to 2.4 MHz in the 1 Lux to 10^5^ Lux illuminance range.

The output signal of each photodetector is sampled by the ADC at a sampling rate of about 2000 samples/s. However, as shown in Figure 13a, the high cut-off frequency of the photoreceptor can be much greater than 1 kHz at high levels of luminosity. To keep the high cut-off frequency of the auto-adaptive circuit of each photodetector constant, an anti-aliasing filter was implemented by cascading a first order low-pass filter based on a gm-C filter [37]. A high capacitance was directly integrated into the pixels, and the transconductance amplifier (OTA) was biased at a very low current level. The resulting cut-off frequency remained constant and equal to 300 Hz, regardless of the illuminance.

At last, as shown in Figure 12, a follower stage based on an operational amplifier was introduced between the anti-aliasing filter and the ADC to reduce the input time constant and, thus, to improve the accuracy required by the sampling rate of 2 ksamples/s.

The third and last layer of metallization was dedicated to power lines. The photodetector layout is compatible with the low resistivity of the power lines, leading to a low dropout voltage along these lines, as well as a good match between pixels.

### 4.2. Characterization of the Auto-Adaptive Photodetectors

**Figure 14.**
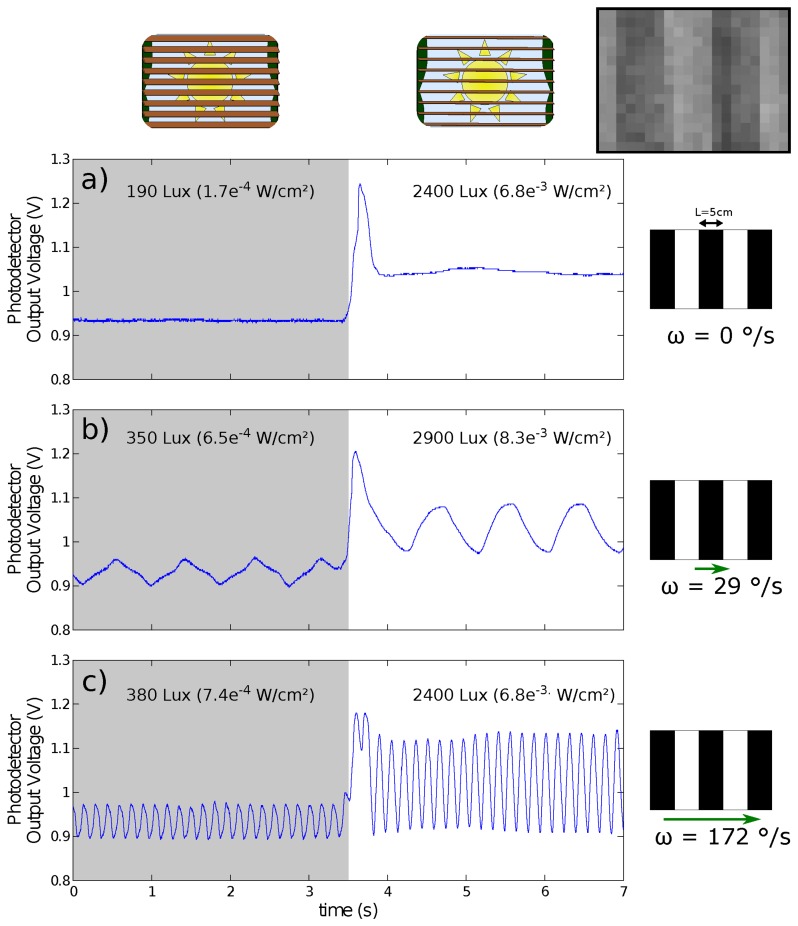
Response of one CurvACE element (a photodetector with optics) to sharp changes in the illuminance (obtained by opening a sun-blind) after digitizing and sampling the data at a frequency of 500 Hz. The photodetector output signal was recorded while facing a periodic pattern (a set of stripes with a width of 50 mm placed 195 mm from the sensor) exposed to natural lighting conditions) (**a**) stationary (static state) and translating at a speed of (**b**) 29°/s and (**c**) 172°/s. The photodetector compensated quickly (about 0.5 s) for the increase in the illuminance and adapted its gain, as well as amplified the transient signals generated by the moving pattern. The inset (upper right corner) shows the periodic pattern acquired by the CurvACE sensor at a distance of 15 cm with a region of interest composed of 20 by 15 ommatidia under a lighting of 1500 Lux.

The auto-adaptive photodetectors were designed to ensure low sensitivity to ambient lighting changes and high sensitivity to the contrasts. Figure 14 shows the dynamic gain adjustment of one CurvACE element (photodetector equipped with optics) facing a set of still or moving strips placed 195 mm from the device. It is obvious that the Delbrück pixel compensates quickly within 400 ms for any sharp change in the illuminance induced by opening a sun-blind manually. Even at low illuminance levels, the signal-to-noise ratio (37.3 dB or 45.3 dB depending on the illuminance level, which was equal to 350 Lux or 380 Lux, respectively) was sufficient for the visual processing algorithms. The noise was measured at an illuminance level of 190 Lux (see Figure 14a).

### 4.3. Imaging Characteristics of the CurvACE Compound Eye

**Figure 15.**
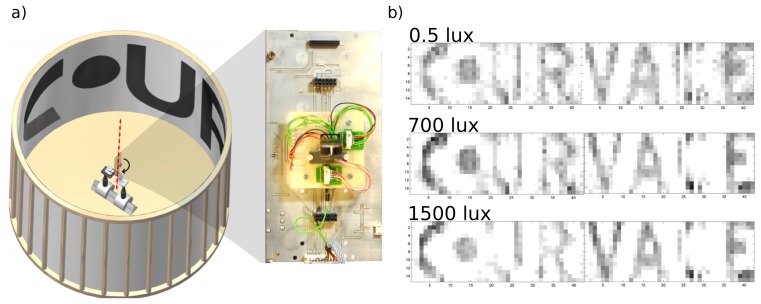
(**a**) The CurvACE device placed in a circular arena (105 cm in diameter) lined with the CurvACE logo. The CurvACE supporting stage rotated around the vertical axis (red dotted line) at an angular speed of 125°/s by means of a stepper motor. (**b**) Images taken from three video sequences acquired by CurvACE under three different lighting conditions: 0.5 Lux, 700 Lux and 1500 Lux. The sensor is able to retain high sensitivity despite the strong differences in the ambient lighting.

Figure 15b shows the image produced by the CurvACE prototype when it was rotating at an angular speed of 125°/s, and the images were acquired at a rate of 25 fps. Despite the relatively coarse resolution, the CurvACE sensor was able to produce images of a sufficiently good quality for the pattern to be recognizable. This experiment was repeated with several levels of background illuminance ranging from 0.5 Lux to 1500 Lux in order to test the ability of the CurvACE sensor to compensate for changes in the illuminance. As shown in Figure 15 and as expected from the experimental data shown in Figure 14, no significant differences were observed between the images obtained.

## 5. Conclusion

The prototype of a compact cylindrically-shaped artificial compound eye was presented, which is suitable for optic flow-based navigation. The well-designed quality of the ommatidia acceptance angles and interommatidial angles lead to a wide, gapless and distortion-free field of view of 180° by 60°. The sampling angle of the 630 ommatidia is about 4.2°. Moreover, it is straightforward to achieve a 360° panorama view by combining two CurvACE devices or two microoptics-CMOS stacks onto an extended flexible PCB. In this paper, we showed, for the first time, the dynamical response at the photoreceptor level (Bode diagram and time response) and provided examples of full resolution images acquired by CurvACE while the latter was stimulated by a moving pattern under different lighting conditions. CurvACE benefits from the use of auto-adaptive photodetectors, which lead to high sensitivity for fast changing contrasts, while adapting to slow variations of background illumination over four decades from 0.5 Lux (moon night) to 1500 Lux (sunny day). An extra low-pass filter was incorporated to maintain a constant cut-off frequency independent of the illuminance level. Another advantage compared to standard CMOS camera sensors is the high sampling rate, which allows a readout of up to 1950 fps. The versatile approach makes use of rigid materials (silicon and glass) and planar assembly technology, which enables high alignment accuracy for established state-of-the-art fabrication processes that could be set-up in a large scale, *i.e.*, micro-optics fabrication, wire bonding and precision cut-off grinding for column separation. Nevertheless, this concept can be adapted to a wide range of shapes, photoreceptor layouts or optical assembly. Such optic flow extracting sensors may find their application in autonomous robots and vehicles. In addition, CurvACE could offer unique opportunities to suggest new explanations for animals' outstanding performances in tasks, such as navigation, obstacle avoidance or chasing and, thus, help to foster the development of novel visual processing algorithms. Future improvements in the design of the compound eye may result in devices with even smaller packages, induced by progress, for example, in CMOS fabrication, processing optical materials or precision cut-off grinding.

## Figures and Tables

**Table 1.** Characteristics of various communication buses, including the one used for the CurvACE readout (in bold), with N the number of *D_out_* signals (equal to 42 for CurvACE) and *M_sync_* the number of sync signals (equal to two for CurvACE).

	**Direct Communication Protocol (DCP)**	UART	SPI	*I^2^C*	CAN	oneWire
Maximum bit rate	**Main quartz of microcontroller**	115 kbits	40 Mbits	400 kbits	1 Mbit	140 kbit
No. of pads per column for readout	**3**	2	4	2	2	1
No. of tracks on the flex PCB	*N\*(*M_sync_*) + *M_sync_* + 1= 24 tracks (2 × 12)	N+1	N+3	2	2	1
Remarks	**Curv ACE choice**	Too slow	Too slow	Requires EEPROM	Very complex	Too slow
